# Hematological Parameters and Occupational Ionizing Radiation Exposure in Interventional Healthcare Workers: A Longitudinal Matched Case-Control Study

**DOI:** 10.3390/epidemiologia7050147

**Published:** 2026-10-21

**Authors:** Gabriele d’Ettorre, Giuseppe Loiacono, Gianmarco Giannelli, Prisco Piscitelli, Giancarlo Ceccarelli

**Affiliations:** 1 Department of Occupational Medicine, Local Health Authority of Lecce, 73100 Lecce, Italy; 2 Department of Experimental Medicine, University of Salento, 73100 Lecce, Italy; 3 Department of Public Health and Infective Diseases, Sapienza University of Rome, 00165 Rome, Italy

**Keywords:** ionizing radiation, healthcare worker, hemogram, occupational safety, radiation protection, longitudinal study

## Abstract

Background/Objectives: The rising volume of fluoroscopy-guided interventions poses an occupational safety challenge for healthcare workers (HCWs) exposed to chronic low-dose ionizing radiation (LDR), which carries stochastic risks of bone marrow suppression and radiation-induced cytopenias. Real-world longitudinal evidence validating the efficacy of modern protection standards on specific peripheral hematological parameters remains limited. This study aims to evaluate the peripheral hemogram profiles of interventional HCWs exposed to LDR, compared to those of a non-exposed control cohort, from baseline (T0) to follow-up (T1). Methods: A longitudinal matched case-control study evaluated the complete blood count with differential leukograms of 136 interventional HCWs exposed to LDR compared to those of 272 matched non-exposed controls from baseline (T0) to follow-up (T1), stratifying by role, seniority, and cumulative ionizing radiation dose. Results: The mean annual radiation dose was 0.22 mSv, and the mean cumulative radiation dose was 4.11 mSv. Hemogram indicators showed no significant differences between cases and controls at T0 and T1 (*p* > 0.05). Stratification by length of service (<10 vs. >10 years), and radiation dose (<3 vs. ≥3 mSv) demonstrated no significant variations in erythrocyte, leukocyte, or platelet counts. Regression analysis confirmed no association between hematological parameters and job seniority or radiation dose. All radiation doses remained below mandatory limits. Conclusions: The findings provide objective evidence of the effectiveness of current safety measures countering radiation-induced hematotoxicity in hospital settings. Longitudinal tracking confirms the absence of adverse hematological shifts, suggesting that structured training and strict personal protective equipment (PPE) use mitigate hematotoxicity risks among interventional staff exposed to ionizing radiation in healthcare settings. Safety protocols should maintain strict PPE compliance and safety training. Occupational health policy needs to prioritize continuous dosimetry monitoring, as routine hematological screening may not show early low-dose exposure effects.

## 1. Introduction

Minimizing the occupational exposure of healthcare workers (HCWs) to ionizing radiation (IR) is a primary objective for health organizations and occupational health systems worldwide [1,2,3]. Continuous advancements in diagnostic and therapeutic procedures using IR have led to an increased number of interventional X-ray procedures where HCWs need to maintain close physical contact with the patient during radiation exposures [4,5,6]. In particular, HCWs performing fluoroscopy-guided interventions are potentially exposed to a chronic risk of biological effects linked to IR [7,8,9]. Occupational exposures of HCWs in hospital settings are usually classified as chronic low-dose radiation (LDR) exposures, as LDR is defined as less than or equal to 100 mSv, and the LDR rate is defined as less than or equal to 6 mSv per hour [10,11,12,13]. Although radiation protection measures are adopted in occupational exposures, radiation epidemiology studies suggest that LDR might still correlate with specific tissue reactions, such as radiation-induced cataracts or long-term cardiovascular diseases secondary to microvascular endothelial dysfunction [14,15,16,17,18,19].

To date, a growing body of literature has highlighted HCWs performing interventional procedures using X-rays as a major source of occupational IR exposure in the healthcare sector, given the complex procedural architectures and lengthy fluoroscopy times [7,17,18]. For regulatory and safety management purposes, radiation protection relies on the optimization principle, asserting that exposures must be kept “as low as reasonably achievable” (ALARA) [20,21,22]. This approach aligns with the linear no-threshold (LNT) model, which presumes a linear and causal relationship between IR and human cancer risk, excluding a safe biological threshold [23]. Although the LNT model remains the international benchmark for safety regulations, its validity at occupational exposure levels is highly debated due to the difficulty of gathering statistically powered epidemiological cohorts capable of isolating LDR effects from baseline population variations [24,25]. Furthermore, complex radiobiological mechanisms—such as the adaptive response, bystander effects, genomic instability, abscopal effects, and inter-individual variations in radiosensitivity—make the issue of LDR risk estimation even more complex, as the available information is limited [26].

Despite these debates, several molecular studies have identified DNA damage pathways associated with LDR exposure, underscoring the need for rigid personal protection and systematic health surveillance of such workers [27,28]. In many European countries, including Italy, the routine analysis of the peripheral hemogram represents a historical cornerstone of occupational health surveillance for HCWs exposed to LDR, aiming to detect early hematological variations induced by myelosuppressive risks [29,30]. Standard surveillance indicators include red blood cell (RBC) count, hemoglobin (Hb), white blood cell (WBC) count, platelet (PLT) count, and absolute or relative lymphocyte (LYM) and monocyte (MO) counts [30]. Monitoring these specific lineages is clinically relevant, as lymphocytes represent the most radiosensitive peripheral cell line, capable of undergoing rapid radiation-induced apoptosis, while shifts in platelet and erythrocyte parameters can signify early-stage bone marrow suppression.

The current literature presents conflicting data regarding the clinical utility and findings of routine hemograms under modern safety protocols. For instance, a case-control study by Qian et al. [31] reported a downward shift in peripheral blood cells consistent with subclinical leukopenia and thrombocytopenia among HCWs exposed to a mean annual effective dose of 1.37 mSv and a mean cumulative dose of 13.7 mSv. In line with these findings, the study performed by Liu et al. [32] described a non-linear dose–response relationship between the cumulative radiation dose and fluctuations in platelet counts, suggesting an unpredictable impact on megakaryopoiesis. Conversely, a comprehensive review conducted by Vaiserman et al. [24] concluded that statistically significant adverse health outcomes are rarely detectable in modern cohorts exposed to standard occupational LDR, noting that historical positive findings were frequently confounded by lifestyle habits like smoking or alcohol consumption.

Given these ongoing debates and the shift toward proactive safety validation, we designed a retrospective longitudinal case-control study. This study aimed to evaluate the peripheral hemogram profiles of interventional HCWs exposed to LDR, compared to those of a non-exposed control cohort, from baseline (T0) to follow-up (T1), while examining whether internal variations exist regarding cumulative X-ray dose or length of service. Rather than merely screening for pathology, this investigation serves as an institutional audit to validate the efficacy of current radioprotection measures against hematologic radiotoxicity. Data were retrieved from the hospital’s Occupational Prevention and Protection Service database, containing standardized medical records and dosimetric history. This study was conducted in accordance with the mandatory health surveillance frameworks dictated by Italian Legislative Decree 101/2020 [33], which transposes European Directive 59/2013 [34].

## 2. Materials and Methods

We performed a matched retrospective cohort study utilizing clinical and occupational data from interventional HCWs exposed to IR, matched to HCWs who were not exposed to IR as controls, employed at the Vito Fazzi Hospital (Lecce, Italy). This study monitored exposed and control HCWs at two distinct time points: baseline (T_0_) and follow-up (T_1_) during their mandatory periodic occupational health surveillance from 1 January 2000 to 31 December 2025. The study population included 136 interventional HCWs occupationally exposed to IR in interventional settings (68 medical doctors and 68 nurses) and 272 non-exposed HCWs as controls (136 medical doctors and 136 nurses). Exposed HCWs were enrolled based on a minimum eligibility criterion of one year of employment in interventional settings. The 136 cases were derived from a total source population of 141 interventional personnel identified within the hospital’s database during the 2000–2025 study timeframe. A total of 5 healthcare workers were excluded from the final cohort because they had less than 1 year of service in interventional settings.

The total duration of observation for the occupationally exposed cohort spanned from a minimum of 1.0 year to a maximum of 26.0 years, yielding a mean follow-up period of 18.9 (±1.25) years.

Personal radiation exposure for the exposed cohort was systematically monitored and quantified using mandatory personal thermoluminescent dosimeters (TLDs). Controls were closely matched for major potential confounding factors—specifically age, biological sex, years of employment, and baseline lifestyle habits (smoking status and alcohol intake)—ensuring that the two groups differed exclusively with respect to occupational IR exposure.

Baseline (T_0_) of exposed HCWs was defined as the time of employment, corresponding to the initial occupational health medical examination prior to IR exposure. Follow-up (T_1_) corresponded to either the cessation of their radiation exposure or, for actively exposed HCWs, the final data collection point at the end of the study period (31 December 2025). A strict 1:2 individual subject-to-subject matching design was employed to pair each exposed cohort subject (n = 136) with two specific unexposed control subjects (n = 272). To preserve this matched triplet structure in the statistical analysis, a unique identifier variable (Stratum_ID, 1–136) was assigned to each specific case-control set and entered as the stratification/clustering factor in all conditional regression models. Exclusion criteria comprised a history of non-occupational radiation exposure, preexisting hematological or immunological disorders, history of myelosuppressive medication, and active infectious diseases at baseline (T_0_) or follow-up (T_1_). All interventional HCWs utilized personal protective equipment (PPE), including lead aprons, thyroid shields, and protective eyewear [15,35,36].

The objective was to determine whether long-term, real-world exposure to LDR under standard operating conditions correlates with alterations in peripheral hemogram indices, including red blood cell (RBC) count, hemoglobin (Hb), white blood cell (WBC) count, platelet (PLT) count, absolute lymphocyte (LYM) count, absolute neutrophil (NEU) count, and absolute monocyte (MO) count. To determine whether long-term, real-world exposure to LDR under standard operating conditions correlates with alterations in peripheral blood parameters, multiple linear regression analyses were performed exclusively within the exposed HCWs cohort. The models targeted the individual longitudinal delta variations (Delta = T_1_ − T_0_) for each of the seven hemogram indices as dependent variables. Total cumulative radiation dose was evaluated as the primary independent predictor, while adjusting for baseline (T_0_) values, age at enrollment, and total follow-up duration (years of work) to control for structural confounding and multicollinearity.

Individual radiation exposure for the interventional HCWs was monitored continuously using personal thermoluminescent dosimeters (TLDs) based on LiF: Mg, Ti chips, worn monthly at the chest level under the protective lead apron. These dosimeters measured the personal dose equivalent at a depth of 10 mm, Hp(10), and were calibrated annually by the institutional Health Physics Service against ISO 4037 reference X-ray beams. The minimum detection limit (MDL) was 0.05 mSv per month, and readings below this threshold were recorded as zero. To account for the non-uniform scatter radiation field inherent to interventional practices and avoid underestimating the exposure of unshielded areas (e.g., head, neck, eye lens), the true whole-body effective dose (E, expressed in mSv) was estimated applying the validated operational single-badge algorithm: E = 0.5 × Hp (10)_under_. Total cumulative radiation dose was calculated using the formula: D = Σ (from i = 1 to n) E_i where D represents the total cumulative radiation dose (mSv), Ei is the individual effective dose recorded during the monitoring interval, and n denotes the total number of surveillance periods from T_0_ to T_1_.

### Statistical Analyses

Data were analyzed using SPSS version 19.0. Descriptive statistics were used to summarize the baseline clinical and demographic characteristics of the cohort, expressed as mean ± standard deviation (SD) for normally distributed variables, median and interquartile range (IQR) for non-normally distributed variables, and frequencies with percentages for categorical data. Data normality was verified utilizing the Shapiro–Wilk test. To account for the 1:2 matched design of the cohort, univariate comparisons between the exposed and unexposed groups were performed using conditional linear regression for continuous variables, and conditional logistic regression for categorical variables. To evaluate the potential impact of occupational exposure on peripheral blood counts, job seniority (years of work) and cumulative IR doses (mSv) were analyzed using a dual approach. For descriptive purposes and to facilitate clinical interpretation, these exposures were stratified as categorical variables: job seniority was dichotomized into short-term (<10 years) and long-term (≥10 years) cohorts, while cumulative radiation dose was divided into low-exposure (<3 mSv) and higher-exposure (≥3 mSv) sub-cohorts based on the median distribution of the exposed sample. Conversely, to maximize statistical power and avoid the information loss inherent in categorization, both job seniority and accumulated radiation dose were entered as continuous variables in the multivariable models evaluating longitudinal changes. To evaluate longitudinal changes, delta values (Δ = T_1_ − T_0_) were calculated for each of the seven peripheral hemogram indicators. Multiple linear regression models were constructed using these delta variations as the dependent variables within the exposed cohort to assess their association with independent exposure metrics, specifically total cumulative radiation dose. These models explicitly included job seniority as a continuous covariate (expressed as total years of work), alongside baseline (T_0_) hematological values and age at enrollment, to control for temporal confounding and minimize information loss. Model-level goodness of fit was evaluated using R^2^, adjusted R^2^, and the F-statistic with its corresponding degrees of freedom (df). Standard regression diagnostics were systematically performed: multicollinearity among predictors was assessed via the Variance Inflation Factor (VIF), while homoscedasticity and normality of residuals were verified using the Breusch–Pagan test and Shapiro–Wilk test, respectively. Crucially, to directly assess whether longitudinal hematological trajectories differed between exposed workers and their matched unexposed peers across the entire study population (n = 408), conditional linear regression models were executed. In these models, the longitudinal delta (Δ) of each hemogram index served as the dependent variable, while occupational exposure status (Exposed vs. Control) was entered as the primary independent predictor, automatically accounting for the 1:2 matched design. To account for multiple hypothesis testing across the seven hematological indices, all *p*-values were adjusted using the Benjamini–Hochberg false discovery rate (FDR) procedure. Statistical significance was set a priori at adjusted *p* < 0.05.

## 3. Results

The clinical and demographic characteristics of the study population demonstrated alignment between cases and controls (Table 1). No significant differences were observed regarding age (cases: 47.9 ± 2.3 vs. controls: 47.6 ± 2.1 years), occupational seniority (cases: 19.3 ± 2.4 vs. controls: 18.9 ± 3.4 years, *p *> 0.05), alcohol consumption, or smoking habits (*p* > 0.05).

The exposed cohort exhibited a mean radiation seniority of 18.9 years (SD 1.25), with an individual observation range between 1.0 and 26.0 years, with 28 workers having less than 10 years of exposure and 108 workers exceeding 10 years. Dosimetric records revealed an average annual effective dose of 0.22 mSv (range: 0.1 to 2.10 mSv/year) and a per capita cumulative effective dose of 4.11 mSv. Stratification by cumulative dose showed that 60 HCWs were exposed to <3 mSv and 76 HCWs to ≥3 mSv. Moreover, all the recorded occupational doses fell well below the regulatory threshold established by Italian Legislative Decree 101/2020 [33] and indicated a low-exposure operating environment.

A primary finding of this longitudinal study is the remarkable stability of the peripheral hemogram over time, highlighting that standard interventional protocols do not induce myelosuppressive variations in the studied HCWs. As detailed in Table 2 and Table 3, there were no statistically significant differences in any hematological indicators between cases and controls (*p* > 0.05). Furthermore, during the entire multi-decade observation period, no interventional healthcare worker required temporary or permanent removal from radiation-exposed work or incurred health-surveillance-related task restrictions due to occupational hematological alterations or suspected bone marrow suppression.

For instance, among cases and controls at T1, the white blood cell counts and platelet counts remained closely matched and entirely within physiological limits.

Subgroup analyses within the exposed cohort confirmed this lack of statistical variance; in fact, no significant differences were identified when comparing hematological profiles between interventional medical doctors and nurses exposed to LDR at T1 (Table 4) (*p* > 0.05). Furthermore, after stratifying cases by occupational seniority (<10 years vs. >10 years; Table 5) or cumulative dose (<3 mSv vs. ≥3 mSv; Table 6) no statistical deviations in cellular lineages (*p* > 0.05) were found. The multi-adjusted regression models revealed no statistically significant independent associations between cumulative radiation exposure and longitudinal variations in any of the analyzed hematological parameters (Table 7). Specifically, after adjusting for potential confounders, the cumulative radiation dose did not show a significant relationship with changes in white blood cell count, red blood cell count, or hemoglobin levels. Similarly, changes in absolute neutrophil and monocyte counts remained stable and unaffected by the cumulative dosimetric workload. Prototypical downward trends that approached borderline significance in the unadjusted models were observed for absolute lymphocyte changes and platelet count variations. However, after applying the Benjamini–Hochberg false discovery rate (FDR) procedure to correct for multiple hypothesis testing across the seven blood metrics, these trends were entirely attenuated, yielding adjusted *p*-values of 0.31 for both parameters. The expanded multivariable regression analyses restricted to the exposed cohort, incorporating full model-level statistics and covariate coefficients, are detailed in the updated Table 7. The predictors accounted for 16% to 28% of the total variance in hematological deltas (with R^2^ ranging from 0.16 to 0.28), driven primarily by the strong anchoring effect of baseline (T_0_) values. Regression diagnostics confirmed the statistical validity of the ordinary least squares (OLS) assumptions: the maximum VIF recorded across all models was 2.45 (well below the conservative threshold of 5.0), demonstrating that simultaneous inclusion of age and job seniority did not induce problematic multicollinearity. Residuals met the assumptions of homoscedasticity (Breusch–Pagan *p* > 0.05) and normality. The direct longitudinal comparison between the exposed group and the matched controls (comprising the total cohort of 408 individuals) is presented in Table 8. The conditional linear regression models revealed that the mean longitudinal delta variations (Δ = T_1_ − T_0_) for erythrocytes, leukocytes, platelets, and all differential leukocyte counts did not differ significantly between interventional healthcare workers and unexposed controls. After applying the Benjamini–Hochberg FDR correction, the exposure status coefficient remained non-significant across all seven blood metrics (adjusted *p* > 0.05), confirming that the long-term hematological trajectory of workers exposed to chronic LDR strictly mirrors that of the non-exposed reference population.

## 4. Discussion

The ongoing increase in fluoroscopy-guided interventions in hospital settings represents a major concern given the long duration of the procedures using IR and the close contact with the patient being treated [35,36]. Although occupational exposures to IR in hospital settings are usually classified as LDR exposures, staff involved in interventional fluoroscopy-guided procedures have been reported to be at potentially significant risk of exposure to radiation levels that may both compromise the hematopoietic system and lead to changes in the peripheral hemogram, even if they use personal protective equipment [35,36]. In the past, a study by Waselenko et al. [37] identified the hematopoietic system of the human body as one of the most sensitive biological indicators in radiobiology; in fact, due to exposure to IR, significant changes may occur in blood cells counts leading to diseases including immunosuppression, infection, inflammation, anemia, coagulation dysfunction, leukemia, myelodysplastic syndrome, and hemophilic cell syndrome.

The primary finding of our study, however, challenges the translation of these risks into clinical practice under modern radioprotection standards. In fact, in our study, interventional HCWs exposed to LDR showed no significant differences in the detected peripheral hemogram indicators compared to the control group. Moreover, no significant differences in such hemogram indicators were found among interventional HCWs compared by seniority and cumulative dose. The comparability of the two groups was further reaffirmed after adjustment for confounders, which did not change the results. This system-wide stability over time strongly suggests that the bone marrow microenvironment does not experience cumulative myelotoxic injury when occupational exposures are strictly kept within the low-dose spectrum.

The present longitudinal study confirms that chronic LDR exposure within current regulatory limits does not induce myelosuppressive variations or alter peripheral hemogram parameters in interventional HCWs. These findings clinically validate the long-term safety and efficacy of standard occupational radioprotection protocols, reassuring healthcare staff regarding the absence of detectable hematotoxicity across different professional roles, seniorities, and cumulative doses.

The findings of our study are consistent with the review conducted by Zare et al. [38] which showed no effect of LDR on the parameters linked to red blood cells (RBC) and hemoglobin, in accordance with other studies that did show any significant changes in the peripheral hemogram after long-term occupational exposures to LDR. This reinforces the concept that, at extremely low occupational doses, homeostatic cellular repair mechanisms and potential radio-adaptive responses effectively counteract the theoretical stochastic risk to blood lineages.

Findings concerning the links between chronic radiation and blood counts are, however, deeply divided in the international literature. Taqi et al. [39], in a case-control study, reported that long-term exposure to LDR in diagnostic radiographers significantly increased the values of both lymphocytes and reactive lymphocytes in all the detected groups compared with the controls. These findings are consistent with the research conducted by Shahid et al. [40] which showed that several hemogram parameters (hemoglobin, white blood cell count, platelet count, hematocrit count, mean corpuscular hemoglobin, mean cell hemoglobin concentration, and neutrophil count) decreased, while only two parameters (red blood cells and lymphocytes) increased after exposure to long-term LDR. Moreover, the study performed by Guo et al. [41] found that, in workers exposed to LDR, the red blood cell counts initially decreased, then increased, and further decreased again in response to the cumulative radiation dose; given these results, the authors hypothesized that mature erythroid progenitor cells in the bone marrow are extremely sensitive to radiation damage, which can lead to extensive red cell death. In fact, previous experimental studies have reported that after total-body irradiation, the erythrocyte count in mice first decreased and then recovered [42,43], which was consistent with the findings in workers exposed to LDR. Moreover, a study by Hosseinabadi et al. [44] showed that the differences in red blood cell and WBC counts in radiation workers before and after exposure were statistically significant, whereas those in platelet counts were not.

In general, despite the lack of consistent epidemiological and experimental data and the disagreement about the impact of LDR on the hemopoietic system, several studies have shown that IR alters hematological parameters [11]. However, as properly highlighted by a recent review conducted by Vaiserman et al. [24], several methodological issues and limitations affected most of the historical epidemiological studies showing the long-term effects of LDR. According to a summary analysis of occupational studies, the authors concluded that statistically significant detrimental health effects of occupational exposure to LDR were not often observed. In several professional groups, these effects can be influenced by confounding factors such as smoking, alcohol consumption, and/or the healthy worker effect (HWE). For example, a common trend observed in the majority of occupational cohorts around the world is that mortality from most causes of death is typically lower in the cohorts exposed to LDR than in the general populations [45,46]. Although such outcomes are likely influenced by the HWE, no quantitative estimations of HWE have been performed, and several authors believe that radiation hormesis induced by LDR could be involved [45,46]. By matching our cases and controls for smoking and alcohol habits (Table 1), our study successfully isolated the radiation variable, validating that the observed “null results” are a true reflection of occupational safety rather than lifestyle confounding factors.

From an occupational health perspective, the findings of our longitudinal case-control study showed that, among the studied HCWs, all the cumulative doses were less than the effective dose limits stated by Italian regulations [33] and led us to hypothesize that, as long as the exposure levels are low and radiation protection methods are used by HCWs, the risk of damage to the hematopoietic system is minimized. In particular, according to growing evidence regarding HCWs’ protection from LDR, the adoption of effective protection methods plays a key role in minimizing both the exposure to IR and the harmful effects of IR [4,47]. In fact, several studies have shown that although interventional HCWs are exposed to IR, decreases in radiation exposure can be achieved by the adoption of preventative measures. According to the ALARA principle, which proposes using the lowest dose possible for sufficient image quality and diagnostic information [19], our findings led us to identify improvement interventions aimed at further minimizing LDR exposures in interventional hospital settings. The absolute hematological safety observed between T0 and T1 provides objective institutional validation that our personal protective equipment (PPE)—including lead aprons (0.5 to 1.0 mm lead equivalence), thyroid shields, and protective eyewear—acts as a robust barrier against scatter radiation. In fact, to date, along with an increase in interventional procedures, the literature cites lax use of interventional installations and failure to use PPE as linked to an increased risk of potential radiation exposure [47,48]. The International Commission on Radiological Protection (ICRP) published practical advice in 2018 to protect all workers involved in interventions [49]. This advice includes exposure monitoring strategies and training, along with recommendations on protective garments and interventional installations. Given the ICRP recommendations and the findings of our study, we implemented training and briefings among interventional HCWs with the aim of further reducing both patient and staff exposure to IR. All measures that protect the patient from unnecessary IR simultaneously reduce the scatter radiation to staff and, therefore, their occupational exposure. These preventative measures focus on, for example, the fluoroscopy time, the number of images, the image frequency, and the stringent use of patient aprons so as to reduce scatter radiation and the associated exposure to the upper body, especially the eyes, thyroid, and head. In particular, according to the ICRP, the use of proper ceiling-suspended shields and table-mounted lead curtains reduces the overall scatter radiation field in the room. However, it must be emphasized that personal protective garments, such as lead aprons, exclusively shield the trunk and the central bone marrow, leaving the operator’s hands entirely exposed to localized scatter radiation during interventional maneuvers [49]. Consistent with the ICRP, the use of a patient apron has to be placed outside the field of the primary beam.

The present study suffers from several limitations. Firstly, the cohort includes only 136 exposed healthcare workers from a single institution, which reduces statistical power and limits the generalizability of the findings; however, the sample size was determined by the total eligible population of interventional HCWs at our hospital, meeting the inclusion criteria during the study period, therefore representing an exhaustive single-center cohort. Secondly, due to the retrospective nature of this registry-based audit, individual compliance with PPE (e.g., consistent use of thyroid shields and eyewear) and the quantitative impact of safety training on daily practice were not tracked or measured. Consequently, while the stable hemogram profiles align with a well-managed protection framework, a direct causal link between training compliance and the null hematological findings cannot be definitively established.

Thirdly, the low mean annual radiation dose (0.22 mSv) confirms excellent safety management but makes it difficult to identify the exact threshold where hematological changes might begin to occur; moreover, standard peripheral blood counts may not be sensitive enough to detect the earliest cellular, sub-cellular, or chromosomal damage caused by chronic low-dose radiation. Fourthly, the study period might not capture the long-term, cumulative stochastic effects of radiation that could take decades to manifest. Fifthly, because our dosimetric tracking relied on a single under-apron TLD badge corrected via a standardized whole-body algorithm, it may not fully capture highly localized, unshielded tissue exposures—such as those involving the thyroid, head, or hands—which are common in prolonged fluoroscopy-guided interventional procedures.

Finally, this is a single-center study: the fact that it was conducted exclusively at the Vito Fazzi Hospital in Lecce limits the external validity (generalizability) of the results, as local standard operating procedures and specific equipment models might differ from other hospital centers.

## 5. Conclusions

Our findings provide objective evidence of the clinical safety and effectiveness of current occupational safety measures aimed at minimizing exposure to IR among interventional HCWs in hospital settings. The implementation of training and briefings for HCWs is expected to further minimize the occupational exposure to IR; however, a longitudinal study is required in order to analyze the effectiveness of such preventative interventions with regard to occupational IR exposure among interventional HCWs. Given that radiation exposure should adhere to the ALARA principle because there is no threshold for the occurrence of stochastic effects of IR, continuous efforts are required to lower the HCWs’ exposure to IR. Ultimately, our findings imply that while routine hemogram screening remains a regulatory mandate under Italian Legislative Decree 101/2020, it lacks the biological sensitivity to serve as an early biomarker for LDR tracking.

Furthermore, these results carry important implications for future medical surveillance in the healthcare sector. They suggest that while routine hematological screening remains a regulatory cornerstone, its predictive focus could be optimized by shifting resources toward more sensitive biological indicators or personalized risk assessments, rather than expecting routine complete blood count alterations in LDR exposure environments. Moreover, future progress should be directed toward individual-level studies using omics-based technologies to detect cellular responses at extremely low doses that are difficult to detect with standard clinical instrumentation.

## Figures and Tables

**Table 1:** Study population.

Variable	Exposed n = 136	Controls n = 272
Age (SD)	47.9 (±2.3)	47.6 (±2.1)
Males	62	124
Females	74	148
Years of work (SD)	19.3 (± 2.4)	18.9 (±3.4)
Years of job exposure to ionizing radiation	18.9 (± 1.2)	0
Alcohol consumption (%)	40 (29.4) *	84 (30.9)
Smoking habit (%)	32 (23.5) *	60 (22.1)

* p > 0.05 compared to control group.

**Table 2:** Comparison of peripheral hematological indices in both exposed and control HCWs at the baseline of this study (T0).

Groups	WBC (×10^9^/L)	RBCs (×10^12^/L)	Hb (g/dL)	PLTs (×10^9^/L)	LYM (×10^9^/L)	NEU (×10^9^/L)	MON (×10^9^/L)
Exposed (n. 136)	6.8 (±1.1) *	4.9 (±0.5) *	13.9 (±1.5) *	249.3 (±48.3) *	2.4 (±0.5) *	2.8 (±0.4) *	0.54 (±0.11) *
Control (n. 272)	6.9 (±1.3)	4.8 (±0.8)	14.1 (±1.3)	251.1 (±42.4)	2.4 (±0.3)	2.7 (±0.7)	0.52 (±0.19)
Exact Raw *p*-value	0.452	0.187	0.192	0.711	0.999	0.114	0.235

* *p* > 0.05 compared to control group.

**Table 3:** Comparison of peripheral hematological indices in both exposed and control HCWs at the end of this study (T1).

Groups	WBC (×10^9^/L)	RBCs (×10^12^/L)	Hb (g/dL)	PLTs (×10^9^/L)	LYM (×10^9^/L)	NEU (×10^9^/L)	MON (×10^9^/L)
Exposed (n. 136)	6.7 (±1.3) *	4.8 (±0.6) *	13.9 (±1.4) *	251.5 (±45.1) *	2.3 (±0.7) *	2.9 (±0.5) *	0.55 (±0.14) *
Control (n. 272)	6.8 (±1.5)	4.8 (±0.8)	13.8 (±1.5)	249.4 (±43.4)	2.3 (±0.8)	2.8 (±0.8)	0.53 (±0.14)
Exact Raw *p*-value	0.518	0.999	0.524	0.651	0.999	0.198	0.182

* *p* > 0.05 compared to control group.

**Table 4:** Comparison of peripheral hematological indices at follow-up (T1) between exposed medical doctors (MDs) and nurses (Ns).

Groups	WBC (×10^9^/L)	RBCs (×10^12^/L)	Hb (g/dL)	PLTs (×10^9^/L)	LYM (×10^9^/L)	NEU (×10^9^/L)	MON (×10^9^/L)
MD (n. 68)	6.8 (±1.8) *	4.9 (±0.7) *	13.8 (±1.5) *	248.1 (±44.9) *	2.4 (±0.5) *	2.8 (±0.9) *	0.54 (±0.13) *
N (n. 68)	6.9 (±1.9)	4.8 (±0.5)	14.1 (±1.3)	251.1 (±42.1)	2.3 (±0.8)	2.9 (±0.6)	0.55 (±0.15)
Exact Raw *p*-value	0.755	0.324	0.218	0.689	0.388	0.441	0.692

* *p* > 0.05 compared to nurses.

**Table 5:** Peripheral hemogram results by length of service among exposed workers at the end of this study (T1).

	<10 Years of Work (n = 28)	≥10 Years of Work (n = 108)	Exact Raw *p*-Value
WBC (×10^9^/L)	6.4 (±1.8) *	6.7 (±1.5)	0.374
RBCs (×10^12^/L)	5.1 (±0.5) *	4.9 (±0.6)	0.108
Hb (g/dL)	13.5 (±1.2) *	13.9 (±1.4)	0.165
PLTs (×10^9^/L)	248.8 (±46.5) *	250.1 (±42.1)	0.884
LYM (×10^9^/L)	2.1 (±0.5) *	2.3 (±0.7)	0.151
NEU (×10^9^/L)	3.1 (±0.4) *	2.8 (±0.5)	0.068
MON (×10^9^/L)	0.51 (±0.12) *	0.52 (±0.14)	0.729

* *p* > 0.05 compared to HCWs with >10 years of work.

**Table 6:** Comparison between cumulative radiation dose and peripheral hemogram among exposed workers at the end of this study (T1).

Cumulative Radiation Dose	WBC (×10^9^/L)	RBCs (×10^12^/L)	Hb (g/dL)	PLTs (×10^9^/L)	LYM (×10^9^/L)	NEU (×10^9^/L)	MON (×10^9^/L)
<3 mSv (n. 60)	6.8 (±1.5) *	4.9 (±0.5) *	13.7 (±1.2) *	248.9 (±45.2) *	2.2 (±0.63) *	2.8 (±0.9) *	0.52 (±0.18) *
≥3 mSv (n. 76)	6.7 (±1.8)	4.7 (±0.6)	14.1 (±1.4)	250.1 (±48.9)	2.3 (±0.7)	2.7 (±1.1)	0.51 (±0.15)
Exact Raw *p*-value	0.728	0.062	0.089	0.881	0.384	0.575	0.725

* *p* > 0.05 compared to cases with cumulative radiation dose ≥ 3 mSv.

**Table 7:** Multiple linear regression models evaluating the impact of cumulative radiation dose on hemogram changes (Δ T1 − T0) among exposed workers (n = 136), with all covariates included.

Hemogram Index (Δ)	Predictor/Covariate	Unstandardized Coeff. (B)	95% Confidence Interval (CI)	Standardized β	t	*p*-Value	Model-Level Statistics & Diagnostics
Δ WBC (×10^9^/L)	Cum. Radiation Dose	−0.012	[−0.034, 0.010]	−0.11	−0.92	0.36	R^2^ = 0.24 Adj R^2^ = 0.22 F(4, 131) = 10.34 Max VIF = 2.45
	Baseline Value (T_0_)	−0.340	[−0.450, −0.230]	−0.42	−5.15	<0.001 *	
	Age at Baseline	0.015	[−0.010, 0.040]	0.08	1.05	0.30	
	Job Seniority	−0.018	[−0.045, 0.009]	−0.09	−1.12	0.26	
Δ RBC (×10^12^/L)	Cum. Radiation Dose	0.004	[−0.008, 0.016]	0.08	0.68	0.25	R^2^ = 0.21 Adj R^2^ = 0.19 F(4, 131) = 8.71 Max VIF = 2.45
	Baseline Value (T_0_)	−0.280	[−0.380, −0.180]	−0.39	−4.80	<0.001 *	
	Age at Baseline	0.002	[−0.005, 0.009]	0.04	0.52	0.60	
	Job Seniority	−0.003	[−0.012, 0.006]	−0.05	−0.61	0.54	
Δ Hb (g/dL)	Cum. Radiation Dose	−0.050	[−0.310, 0.210]	−0.04	−0.39	0.35	R^2^ = 0.26 Adj R^2^ = 0.24 F(4, 131) = 11.50 Max VIF = 2.45
	Baseline Value (T_0_)	−0.310	[−0.420, −0.200]	−0.44	−5.32	<0.001 *	
	Age at Baseline	0.012	[−0.022, 0.046]	0.06	0.71	0.48	
	Job Seniority	−0.015	[−0.048, 0.018]	−0.07	−0.88	0.38	
Δ PLT (×10^9^/L)	Cum. Radiation Dose	−0.240	[−0.580, 0.100]	−0.18	−1.40	0.09	R^2^ = 0.19 Adj R^2^ = 0.17 F(4, 131) = 7.68 Max VIF = 2.45
	Baseline Value (T_0_)	−0.290	[−0.400, −0.180]	−0.36	−4.48	<0.001 *	
	Age at Baseline	0.180	[−0.220, 0.580]	0.05	0.59	0.56	
	Job Seniority	−0.210	[−0.620, 0.200]	−0.06	−0.74	0.46	
Δ LYM (×10^9^/L)	Cum. Radiation Dose	−0.018	[−0.041, 0.005]	−0.21	−1.61	0.07	R^2^ = 0.28 Adj R^2^ = 0.26 F(4, 131) = 12.73 Max VIF = 2.45
	Baseline Value (T_0_)	−0.350	[−0.460, −0.240]	−0.45	−5.44	<0.001 *	
	Age at Baseline	0.003	[−0.008, 0.014]	0.05	0.62	0.54	
	Job Seniority	−0.004	[−0.015, 0.007]	−0.06	−0.77	0.44	
Δ NEU (×10^9^/L)	Cum. Radiation Dose	−0.003	[−0.030, 0.024]	−0.02	−0.22	0.41	R^2^ = 0.23 Adj R^2^ = 0.21 F(4, 131) = 9.78 Max VIF = 2.45
	Baseline Value (T_0_)	−0.320	[−0.430, −0.210]	−0.41	−5.02	<0.001 *	
	Age at Baseline	0.011	[−0.015, 0.037]	0.06	0.74	0.46	
	Job Seniority	−0.014	[−0.042, 0.014]	−0.08	−0.93	0.35	
Δ MON (×10^9^/L)	Cum. Radiation Dose	0.001	[−0.017, 0.019]	0.01	0.11	0.46	R^2^ = 0.16 Adj R^2^ = 0.14 F(4, 131) = 6.23 Max VIF = 2.45
	Baseline Value (T_0_)	−0.260	[−0.360, −0.160]	−0.35	−4.25	<0.001 *	
	Age at Baseline	0.001	[−0.003, 0.005]	0.03	0.34	0.73	
	Job Seniority	−0.001	[−0.004, 0.002]	−0.04	−0.42	0.67	

Note: Models are adjusted for baseline (T_0_) values, age at baseline, and job seniority (entered as a continuous variable in years of work). F-statistic degrees of freedom are structured as df1 = 4 (number of predictors) and df2 = 131 (N − K − 1). * *p *< 0.001 indicates statistical significance for baseline anchors. Benjamini–Hochberg FDR correction attenuated the unadjusted dose effect for LYM and PLT to a stable adjusted *p*-value of 0.31.

**Table 8:** Conditional linear regression assessing the impact of exposure status (exposed vs. matched controls) on longitudinal hematological deltas (Δ = T1 − T0) across the total study population (n = 408).

Hematological Delta (Δ)	Mean Δ Exposed (n = 136)	Mean Δ Controls (n = 272)	Exposure Status Coefficient (B)	95% Confidence Interval (CI)	t	Raw *p*-Value	Adjusted *p*-Value (FDR)
Δ WBC (×10^9^/L)	−0.10	−0.10	−0.004	[−0.024, 0.016]	−0.42	0.67	0.78
Δ RBC (×10^12^/L)	−0.10	0.00	−0.002	[−0.010, 0.006]	−0.51	0.61	0.78
Δ Hb (g/dL)	0.00	−0.30	0.012	[−0.045, 0.069]	0.45	0.65	0.78
Δ PLT (×10^9^/L)	2.20	−1.70	0.350	[−0.210, 0.910]	1.22	0.22	0.51
Δ LYM (×10^9^/L)	−0.10	−0.10	−0.008	[−0.024, 0.008]	−0.98	0.33	0.58
Δ NEU (×10^9^/L)	0.10	0.10	0.002	[−0.012, 0.016]	0.28	0.78	0.78
Δ MON (×10^9^/L)	0.01	0.01	0.001	[−0.005, 0.007]	0.31	0.76	0.78

Note: The reference category for the exposure status variable is ‘Controls’ (0 = control, 1 = exposed). The models utilize conditional linear regression to perfectly control for the structural 1:2 frequency-matched design. Adjusted *p*-values are calculated using the Benjamini–Hochberg false discovery rate (FDR) procedure. No comparisons reached statistical significance (*p* > 0.05).

## Data Availability

The data presented in this study are available on request from the corresponding author.

## Abbreviations

The following abbreviations are used in this manuscript:

IR	Ionizing radiation
MD	Medical doctor
N	Nurse
HCW	Healthcare worker
LDR	Low-dose radiation
ALARA	As low as reasonably achievable
LNT	Linear no-threshold
HWE	Healthy worker effect
RBC	Red blood cell
Hb	Hemoglobin
WBC	White blood cell
PLT	Platelet
LYM	Lymphocyte
NEU	Neutrophil
MO	Monocyte
